# Supplementary material for: Co-Overexpression of TWIST1-CSF1 Is a Common Event in Metastatic Oral Cancer and Drives Biologically Aggressive Phenotype
Source: Cancers (Basel). 2021 Jan 5;13(1):153. doi: 10.3390/cancers13010153 (PMC7795342; doi:10.3390/cancers13010153)
Supplement: Supplementary file 1 [file cancers-13-00153-s001.pdf]

Supplementary Materials

Co-Overexpression of TWIST1-CSF1 is a Common Event in Metastatic Oral Cancer and Drives Biologically Aggressive Phenotype

Sabrina Daniela da Silva, Fabio Albuquerque Marchi, Jie Su, Long Yang, Ludmila Valverde, Jessica Hier, Krikor Bijian, Michael Hier, Alex Mlynarek, Luiz Paulo Kowalski and Moulay A. Alaoui-Jamali

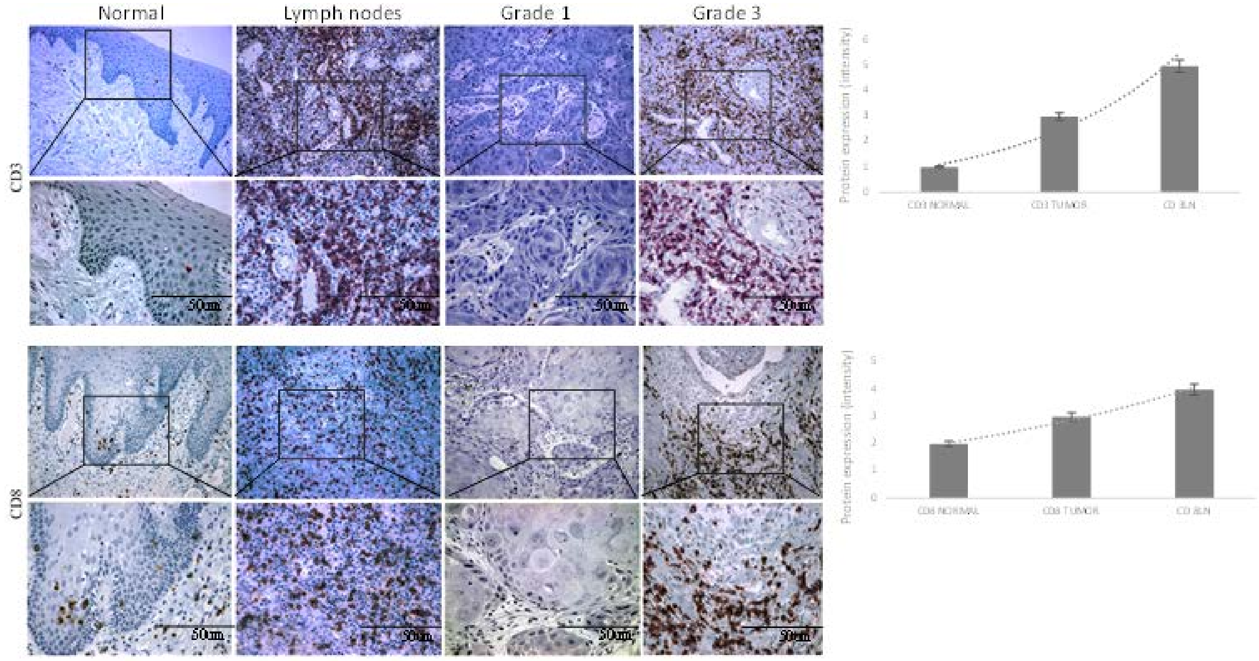

Figure S1. CD3 and CD8 infiltration in tissue samples from patients with OSCC. Immunohistochemistry images for CD3 (**A**) and CD8 (**B**) proteins in morphologically normal epithelial (left side), lymph nodes, and oral cancer at grade 1 and grade 3 samples (right side). A weak staining was observed in morphologically normal epithelial cells while a strong intensity of nuclear immunostaining was detected in oral cancer samples, specially the oral cancer grade 3 tumors and lymph nodes. Graphs (left) represent the CD3 and CD8 immunohistochemistry level (intensity) in normal, tumor and lymph nodes. Original magnification: 20× (top) and 200× (bottom).

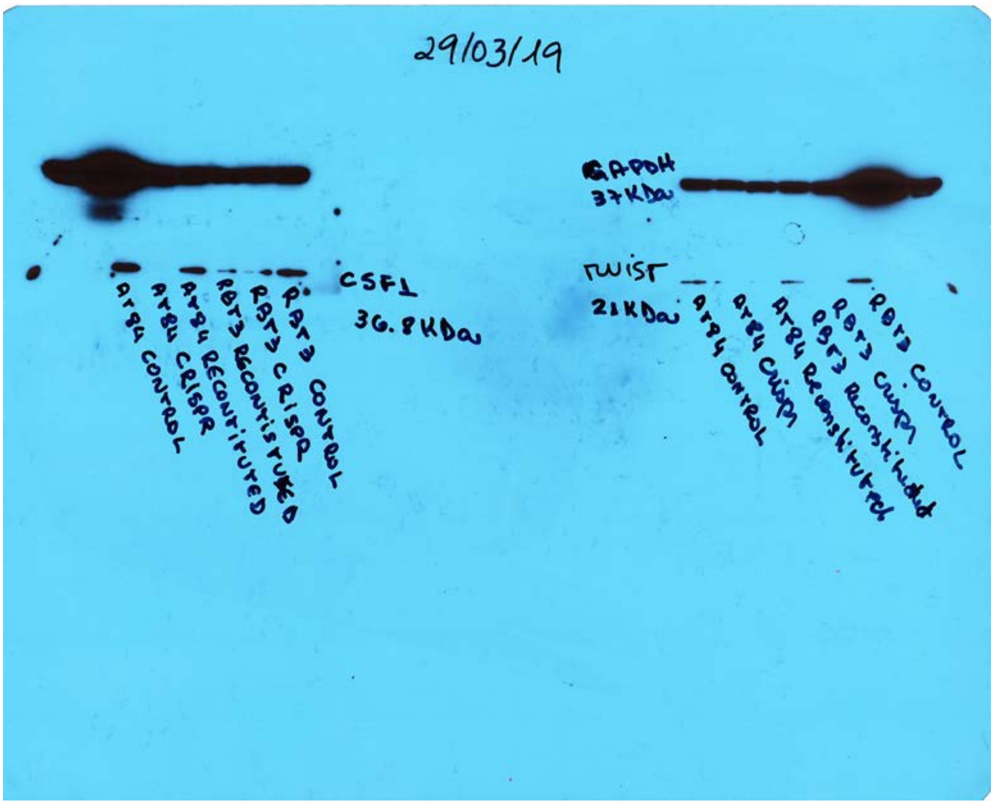
29/03/19
GAPDH 37KDa
CSF1
36.8KDa
AT84 CONTROL
AT84 CRISPR
AT84 RECONSTITUTED
RBT3 RECONSTITUTED
RBT3 CRISPR
RBT3 CONTROL
TWIST
21KDa
AT84 Control
AT84 Crispr
AT84 Reconstituted
RBT3 Reconstituted
RBT3 Crispr
RBT3 CONTROL

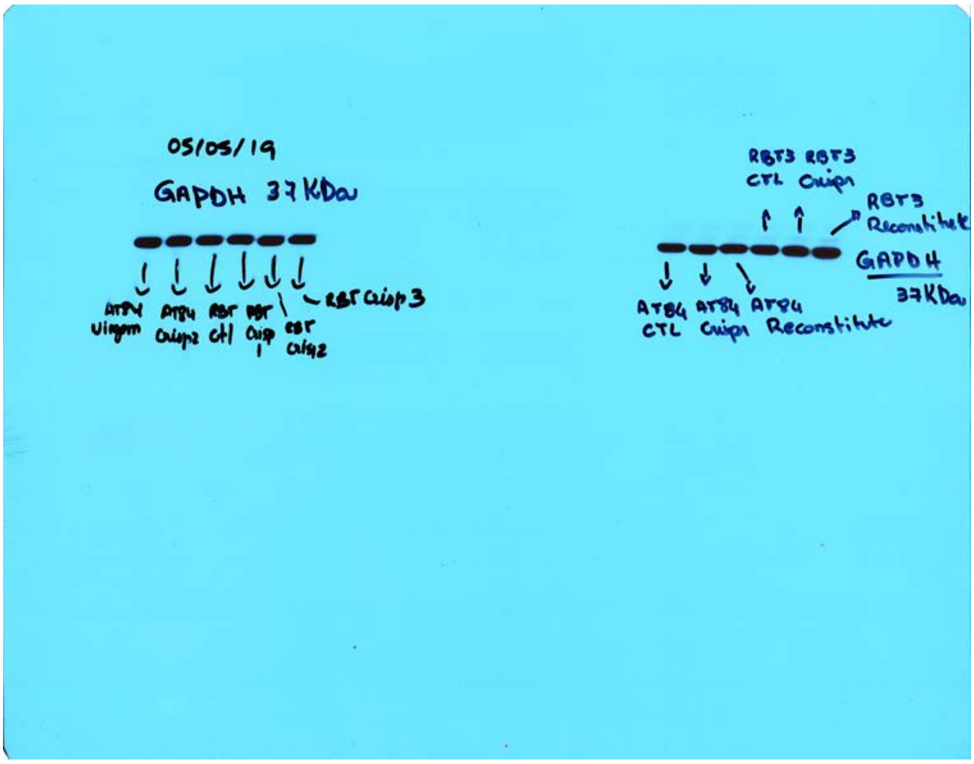
05/05/19
GAPDH 37KDa
AT84 Virgem
AT84 Crisp2
RBT Ctl
RBT Crisp 1
RBT Crisp2
RBT Crisp 3
RBT3 CTL
RBT3 Crispr
RBT3 Reconstitute
GAPDH
37KDa
AT84 CTL
AT84 Crispr
AT84 Reconstitute

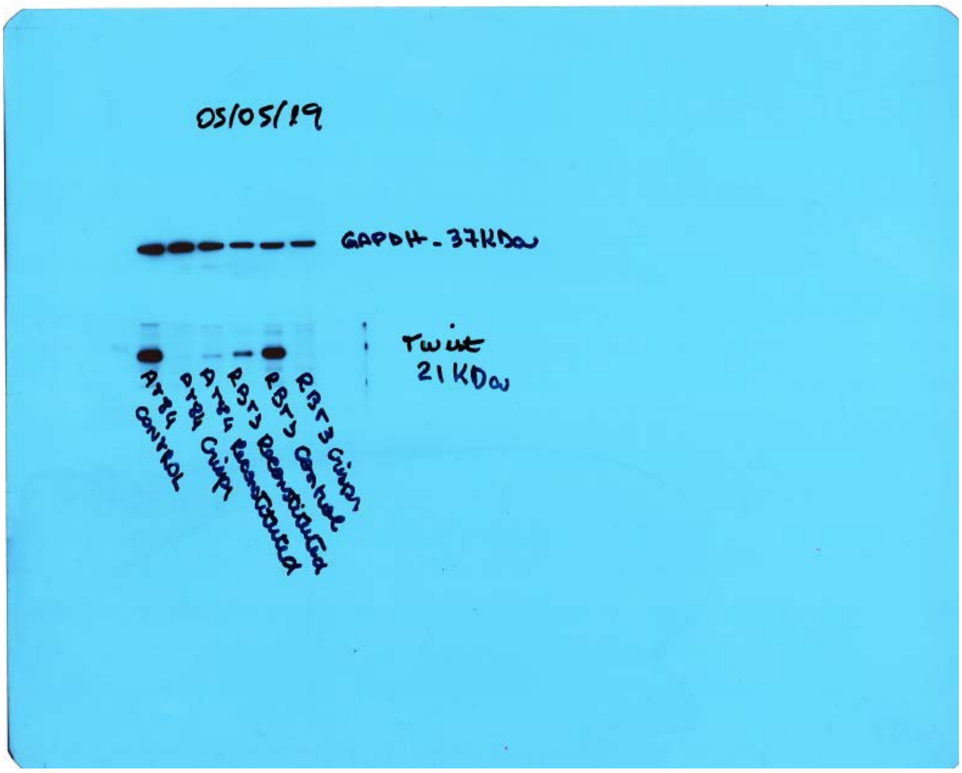

Figure S2. Detailed information about the western blot.

Table S1. Distribution of the OSCC cases according to demographic, lifestyle, and clinical variables.

Variable	Category	Fresh Samples *n* (%)*		Paraffin-Embedded Samples *n* (%)*	
		Non-Metastatic	Metastatic	Non-Metastatic	Metastatic
**Age**	<55 year	5 (50)	2 (20)	39 (54.2)	15 (53.6)
	≥55 year	5 (50)	8 (80)	33 (45.8)	13 (46.4)
Gender	Male	7 (70)	10 (100)	56 (77.8)	25 (89.3)
	Female	3 (30)	0	16 (22.2)	3 (10.7)
Smoking habit	No	4 (40)	2 (20)	7 (10.8)	1 (5)
	Yes	6 (60)	8 (80)	58 (89.2)	19 (95)
Alcohol consumption	No	3 (30)	1 (10)	16 (24.6)	2 (10)
	Yes	7 (70)	9 (90)	49 (75.4)	18 (90)
T stage	T1 + T2	9 (90)	4 (40)	28 (39.4)	5 (22.7)
	T3 + T4	1 (10)	6 (60)	43 (60.6)	17 (77.3)
Nodal status	N0	9 (90)	3 (30)	45 (63.4)	1 (4.3)
	N+	1 (10)	7 (70)	26 (36.6)	22 (95.7)
Tumor grade	I	4 (40)	0	25 (37.3)	14 (24.1)
	II	6 (60)	0	29 (43.3)	28 (48.3)
	III	0	10 (100)	13 (19.4)	16 (27.6)
Recurrence or metastasis	No	10 (100)	0	72 (100)	0
	Yes	0	10 (100)	0	28 (100)
Death	No	10 (100)	6 (60)	43 (59.7)	6 (21.4)
	Yes	0	4 (40)	29 (40.3)	22 (78.6)

* Percentages considering cases with complete information.

Table S2. Association between CSF1 and clinicopathological characteristics.

Variable	Category	CSF1			TWIST1		
		Negative	Positive	*P* Value	Negative	Positive	*P* Value
Age (median)	< 66 year	20 (51.3)	9 (69.2)	0.259	14 (42.4)	12 (52.2)	0.472
	≥ 66 year	19 (48.7)	4 (30.8)		19 (57.6)	11 (47.8)	
Gender	Male	24 (61.5)	7 (53.8)	0.624	23 (69.7)	15 (65.2)	0.724
	Female	15 (38.5)	6 (46.2)		10 (30.3)	8 (34.8)	
Smoking habit	No	18 (46.2)	5 (38.5)	0.629	2 (6.9)	3 (13.6)	0.423
	Yes	21 (53.8)	8 (61.5)		27 (93.1)	19 (86.4)	
Alcohol consumption	No	12 (30.8)	3 (23.1)	0.596	7 (24.1)	6 (27.3)	0.799
	Yes	27 (69.2)	10 (76.4)		22 (75.9)	16 (72.7)	
T stage	T1+T2	**27 (69.2)**	**5 (38.5)**	**0.048**	15 (46.9)	10 (43.5)	0.082
	T3+T4	**12 (30.8)**	**8 (61.5)**		17 (53.1)	13 (56.5)	
Lymph nodes	N0	32 (82.1)	8 (61.5)	0.128	**31 (96.9)**	**7 (30.4)**	**0.005**
	N+	7 (17.9)	5 (38.5)		**1 (3.1)**	**16 (69.6)**	
Tumor recurrence	No	31 (79.5)	9 (69.2)	0.342	**31 (93.9)**	**14 (60.9)**	**0.002**
	Yes	7 (17.9)	5 (38.5)		**2 (6.1)**	**9 (41.1)**	
Status	Alive	**35 (89.7)**	**3 (23.1)**	**0.021**	**26 (78.8)**	**7 (30.4)**	**0.003**
	Died	**4 (10.3)**	**10 (76.9)**		**7 (21.2)**	**16 (69.6)**	

* Percentages considering cases with complete information.

Publisher's Note: MDPI stays neutral with regard to jurisdictional claims in published maps and institutional affiliations.

© 2021 by the authors. Licensee MDPI, Basel, Switzerland. This article is an open access article distributed under the terms and conditions of the Creative Commons Attribution (CC BY) license (http://creativecommons.org/licenses/by/4.0/).
